# Application of affinity capillary electrophoresis for charge heterogeneity profiling of biopharmaceuticals

**DOI:** 10.1002/elps.201900233

**Published:** 2019-10-08

**Authors:** Andrei Hutanu, Steffen Kiessig, Andrea Bathke, Rolf Ketterer, Sonja Riner, Jan Olaf Stracke, Markus Wild, Bernd Moritz

**Affiliations:** ^1^ F. Hoffmann‐La Roche Ltd Basel Switzerland

**Keywords:** Affinity capillary electrophoresis, Charge heterogeneity testing, Flow‐through partial filling, Monoclonal antibody co‐formulation

## Abstract

Charge heterogeneity profiling is important for the quality control (QC) of biopharmaceuticals. Because of the increasing complexity of these therapeutic entities [1], the development of alternative analytical techniques is needed. In this work, flow‐through partial‐filling affinity capillary electrophoresis (FTPFACE) has been established as a method for the analysis of a mixture of two similar monoclonal antibodies (mAbs). The addition of a specific ligand results in the complexation of one mAb in the co‐formulation, thus changing its migration time in the electric field. This allows the characterization of the charged variants of the non‐shifted mAb without interferences. Adsorption of proteins to the inner capillary wall has been circumvented by rinsing with guanidine hydrochloride before each injection. The presented FTPFACE approach requires only very small amounts of ligands and provides complete comparability with a standard CZE of a single mAb.

**Abbreviations**
Ag: antigen
CQA: critical quality attribute
EACA: ε‐amino‐caproic acid
FTPFACE: flow‐through partial‐filling affinity capillary electrophoresis
Gdn: guanidine
GMP: good manufacturing practice
QC: quality control
TETA: triethylenetetramine

## 1 Introduction

A diverse population of charged species in monoclonal antibodies (mAbs) is observed during quality control (QC) of biotechnological pharmaceuticals. These heterogeneities are caused by enzymatic processes as well as spontaneous chemical events 2. Differently charged isoforms of a mAb may have a strong impact on pharmacokinetics or binding properties 3. Hence, health authorities consider charge heterogeneity profiling of biopharmaceuticals as important for product characterization and product stability assessment. For this purpose, different analytical tools for charge heterogeneity profiling, including IEC 4, cIEF 5, and CZE 6 are available. Since these techniques utilize different separation principles, their performance may depend on the individual characteristics of the investigated pharmaceutical and then requires a project specific method selection.

In 2010, He and colleagues published a CZE method which is able to analyze mAbs in a bare fused silica capillary 7. An improved version was published one year later 8. The BGE of this method contains a high concentration of the zwitterionic ε‐amino‐caproic acid (EACA) and the additive triethylenetetramine (TETA). These compounds prevent excessive Joule heating, reduce EOF and dynamically coat the inner capillary wall. Further investigations with different mAbs have demonstrated the generic applicability of this method and have shown some important parameters for the development of tailor‐made adaptions 9. An international cross‐laboratory study has demonstrated that CZE is a simple, fast, reliable, and reproducible method for the relative quantification of different charge species 6. For pharmaceutical applications, the identification of charged species is required as well. For this purpose, a CZE–CZE–MS approach was published recently 10. Due to the individual properties of different biopharmaceuticals, various modifications of the initial method from He and colleagues 8 have been published 11, 12, 13. Goyon et al. demonstrated an alternative approach for the analysis of charge variants of mAbs using a BGE containing polyethylene oxide for dynamic coating and Bis‐tris as a buffering agent. In comparison with the method involving EACA and TETA, separation of additional charged variants for some mAbs was shown but analysis times were prolonged 5‐fold 14.

Due to the increasing complexity of modern biopharmaceuticals, existing methods for characterization reach their limits 1, 15. Therefore, new approaches for the analysis of these sophisticated drugs are highly desirable. ACE represents a synergistic approach combining CZE separation and affinity interaction with antigens, which enables an increased specificity for desired charge species. By means of the addition of a ligand to the separation system, a complex is formed which has different migrating properties than the target itself. This technique was first used in 1992 for the estimation of binding constants between proteins and their ligands 16. It can also be used to examine further parameters of binding reactions (e.g. concentration, stoichiometry, and equilibrium) and the process of complex formation 17, 18, 19.

The set‐up of an ACE experiment strongly depends on the analytes and their binding constants. For tight binding events (K_d_ in the nanomolar range) a pre‐incubation of the protein analyte and its antigen before CZE separation is suitable (equilibrium‐mixture analysis). The formed inert complex will be stable during the analysis, enabling usual CZE conditions. Less stable complexes (K_d_ in the micromolar range) will lead, due to ongoing dissociation during the analysis, to broad zones, in which the equilibrium between ligand and protein is permanently disturbed. A suitable approach for such unstable complexes is the addition of the ligand to the BGE which enables a stable equilibrium inside the capillary due to a constant ligand concentration (mobility‐shift analysis) 17, 18. Alternatively, protein and ligand are injected separately into the capillary (two plug approach). The binding reaction then takes place, when one zone migrates through the other. This requires different electrophoretic mobilities of protein and ligand, so that one will catch up the other. Electrophoretically mediated microanalysis 20, 21, 22 or flow‐through partial‐filling (FTPF) ACE 23, are other names for this methodology introduced in 1992 by Bao et al. 24. The benefit of this method is the use of the capillary as a micro‐reactor. This can minimize the consumption of scarce analytes and provides more options for automation of the analytical procedure. Reaction conditions within this in‐capillary micro‐reactor can be regulated by the variation of parameters like temperature and injection plug length 25.

In this work, we extend the use of flow‐through partial‐filling affinity capillary electrophoresis (FTPFACE) to the charged heterogeneity profiling of complex mAb mixtures (co‐formulations) that are considered to be new remedies for complex diseases like cancer 26. By improved in‐capillary rinsing procedures between the separations and by an optimized three‐plug BGE/analyte/ligand injection protocol the method became very robust and thus suitable for the GMP QC (good manufacturing practice QC) release and stability testing by charge heterogeneity profiling. Therefore, the improved method may contribute to the QC of complex biotech pharmaceuticals for human use.

## 2 Materials and methods

Acetic acid (Cat. no. 33209), EACA (Cat. no. A2504), guanidine hydrochloride (Cat. no. 50940) sodium phosphate monobasic monohydrate (Cat. no. 71507), and TETA (Cat. no. 90460) were purchased from Sigma‐Aldrich/Merck KGaA (Darmstadt; Germany). Acetonitrile (Cat. no. 100030), ethanol (Cat. no. 100983), hydrochloric acid (Cat. no. 109975), methanol (Cat. no. 106007), 2‐propanol (Cat. no. 109634), sodium *n*‐dodecyl sulfate solution (Cat. no.428018), and sodium hydroxide solution (Cat. no. 109138) were from Merck Millipore/Merck KGaA (Darmstadt; Germany). PBS (10x) (Cat. no. 11666789001) was obtained from F. Hoffmann La Roche (Mannheim; Germany). Water of HPLC grade was prepared an a Milli‐Q‐Station (Merck Millipore/Merck KGaA; Darmstadt; Germany) Solutions were filtered through 0.2 µm membrane filters (Corning; New York; USA). Monoclonal antibodies and their corresponding antigens were obtained internally (F. Hoffmann La Roche; Basel; Switzerland). Molecular weight: mAb1≈150 kDa; mAb2≈150 kDa; mAb1Ag≈100 kDa; mAb2Ag≈84 kDa. Isoelectric point: mAb1≈8.5; mAb2≈8.5; mAb1Ag≈6.9; mAb2Ag≈6.7.

CZE separations were performed using a SCIEX PA800plusSystem (Brea; USA) equipped with an UV detector, a 214 nm filter (Cat. no. 144437; SCIEX), a temperature controlled auto sampler (±2°C), and a 30 kV power supply. Experiments were carried out in fused silica capillaries from Molex (Lisle; USA) with i.d. of 50 µm, 20 cm length from the inlet to the detection window and a total length of 30 cm at a temperature of 20°C. During a sequence, samples were stored in the auto sampler at 10°C. For the separation buffer a solution of 400 mM EACA and 2 mM TETA was prepared. The pH was adjusted to 5.7 ± 0.05 with acetic acid using a SevenExcellence pH/Ion meter S500 (Mettler Toledo; Columbus; USA). In the last step, hydroxypropyl methylcellulose was added from a 1% stock solution to achieve a final concentration of 0.05%. Before each injection, capillaries were flushed with rinsing agent (see Table 1) for 10 min; 1 min H_2_O and equilibrated for 1 min with separation buffer (400 mM EACA, 2 mM of TETA, and 0.05% hydroxypropyl methylcellulose). All rinsing steps were performed at 60 psi. For FTPFACE antigens were injected first (0.5 psi for 10 s), followed by the mAb (10 mg/mL) plug (0.3 psi for 3 s) and BGE (0.3 psi for 3 s). For standard CZE without FTPFACE mAb samples were injected using a pressure of 0.3 psi for 3 s. Fresh capillaries were equilibrated for separation buffer conditions by five standard CZE runs with a mAb sample. Polarity was positive (capillary inlet) to negative (capillary outlet) with separation voltage set at +20 kV. The currents observed under the described conditions ranged from 20 to 25 µA. Instrument control, data acquisition and data evaluation were performed with 32 Karat 10.1 software (SCIEX; Brea; USA).

**Table 1:** Summarized results of the rinsing with different agents. After mAb1 injections, mAb1Ag was injected 10–120 times (depending on performance of the rinsing agent). The first injection of mAb1 after the mAb1Ag injections was compared with the control before the antigen injections. Rinsing was performed with each agent for 10 min at 60 psi before each injection in all steps

Agent	Concentration	Effect
HCl	0.1 M	None
	0.5 M	None
	1 M	None
NaOH followed by HCl	0.1 M	None
	0.5 M	Resolution loss in comparison to HCl rinse
	1 M	Resolution loss in comparison to HCl rinse
SDS in Na_2_HPO_4_ pH 7.4	0.05 M 0.01 M	Resolution loss in comparison to HCl rinse
Methanol in H_2_O	10–40% v/v	Basic peak detectable after 10 injections with a lower intensity than control
Ethanol in H_2_O	10–40% v/v	Basic peak detectable after 10 injections with a lower intensity than control
2‐Propanol in H_2_O	10–40% v/v	Basic peak detectable after 10 injections with a lower intensity than control
Acetonitril in H_2_O	10–40% v/v	Basic peak detectable after 10 injections with a lower intensity than control
PBS^a^	1x; ‐ 10x	Reproducible results after 10 antigen injections Basic peak gets lost after 20 antigen injections
Guanidine Chloride (GdnCl) in PBS	0.5 M; 2 M 5x	Reproducible results after 20 antigen injections best results with 0.5 M GdnCl Basic peak gets lost after 50 antigen injections
GdnCl In Na_2_HPO_4_	1 M–1.4 M 0.05 M	Reproducible results after 36 antigen injections best overall results with 1.2 M GdnCl Basic peak gets lost after 120 antigen injections
Guanidine thiocyanate (GdnSCN) in Na_2_HPO_4_	1 M–1.4 M 0.05 M	Reproducible results after 30 antigen injections best results with 1.2 M GdnSCN Basic peak gets lost after 120 antigen injections
Guanidine sulfate (GdnSO_4_) in Na_2_HPO_4_	1.2 M 0.05 M	Reproducible results after 30 antigen injections Basic peak gets lost after 120 antigen injections

a 10x PBS refers to 80 g NaCl, 2 g KCl, 26.8 g Na_2_HPO_4_‐7H_2_O and 2.4 g KH_2_PO_4_ per liter; pH 7.4

## 3 Results and discussion

**Figure 1:**
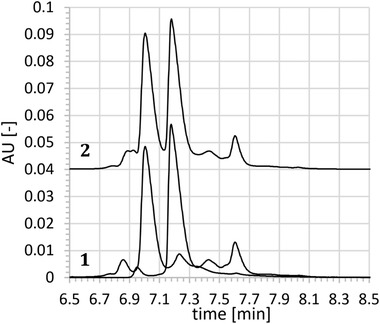
Overlapping charge profiles of mAb1/mAb2 co‐formulation. The lower two lines (1) represent single injections of mAb1 (right) and mAb2 (left) at a concentration of 3 mg/mL. Line 2 illustrates the co‐formulation at 6 mg/mL (3 mg/mL for each mAb). For a better comparison of the peak profiles, the x‐axis has been rescaled.

Antibody co‐formulations are of increasing importance for the pharmaceutical industry since they turned out to be promising remedies against complex diseases like cancer 26. This motivates the development of powerful analytical tools that can reliably and specifically address critical quality attributes (CQAs) of these antibody mixtures. Charge species, if present, are one important group of these CQAs. Therefore, less common but very efficient tools for analytical QC testing of charge species like ACE arouse our interest. The investigation presented here focused on separating two co‐mixed mAbs with similar mass (≈145 kDa), pI (≈8.5) and similar complementarity‐determining regions by ACE. This difficult sample was regarded as some kind of worst case for method development that may allow some generic applicability of the obtained method to other co‐formulations as well. Whilst the main peaks of this co‐mix could be resolved by standard CZE, the minor charge variants of mAb1 and mAb2 are overlapping (Fig. 1). Evaluation of mAb specific profiles is required in order to be specific for all charge species, especially if they turn out to be CQAs.

It has been shown that the BGE used here is close to the optimum for charge heterogeneity profiling of mAbs 8, 9. However, peak deteriorations during initial experiments conducted with a mixture of antibody and antigen (data not shown) led to the assumption that there is a strong capillary wall adhesion of the antigen under separation conditions as results were similar to other publications on this problem 27. In order to address this issue, optimized rinsing procedures should remove proteins from the inner capillary wall as the key to robust method performance and would ideally not influence the separation itself. Therefore, in a first step focus was laid on the capillary washing steps.

### 3.1 Rinsing procedure development

**Figure 2:**
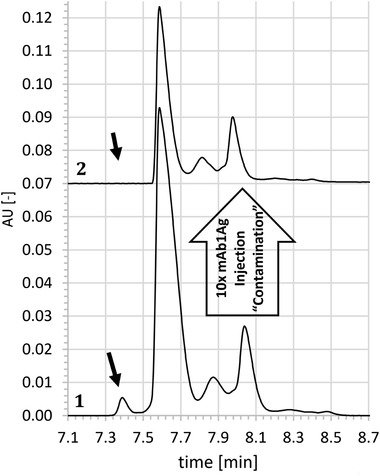
Assay design to detect inner capillary wall adhesion of antigens. Rinsing was performed prior to each injection with 0.1 M HCl for 10 min. Step 1: mAb1 is injected at least five times in a fresh capillary in order to equilibrate the capillary and as a reference (line 1). Step 2: At least ten injections with mAb1Ag are performed. This step should “contaminate” the capillary with mAb1Ag, resulting in a changed peak pattern (indicated by the black arrow in line 1 and 2) for the following runs with mAb1 (step 3; line 2). All runs are perfomed with the same rinsing procedure, so that the effect of a new rinsing agent can be detected by comparing step 1 and step 3 separations. For a better comparison of the peak profiles, the x‐axis has been rescaled.

**Figure 3:**
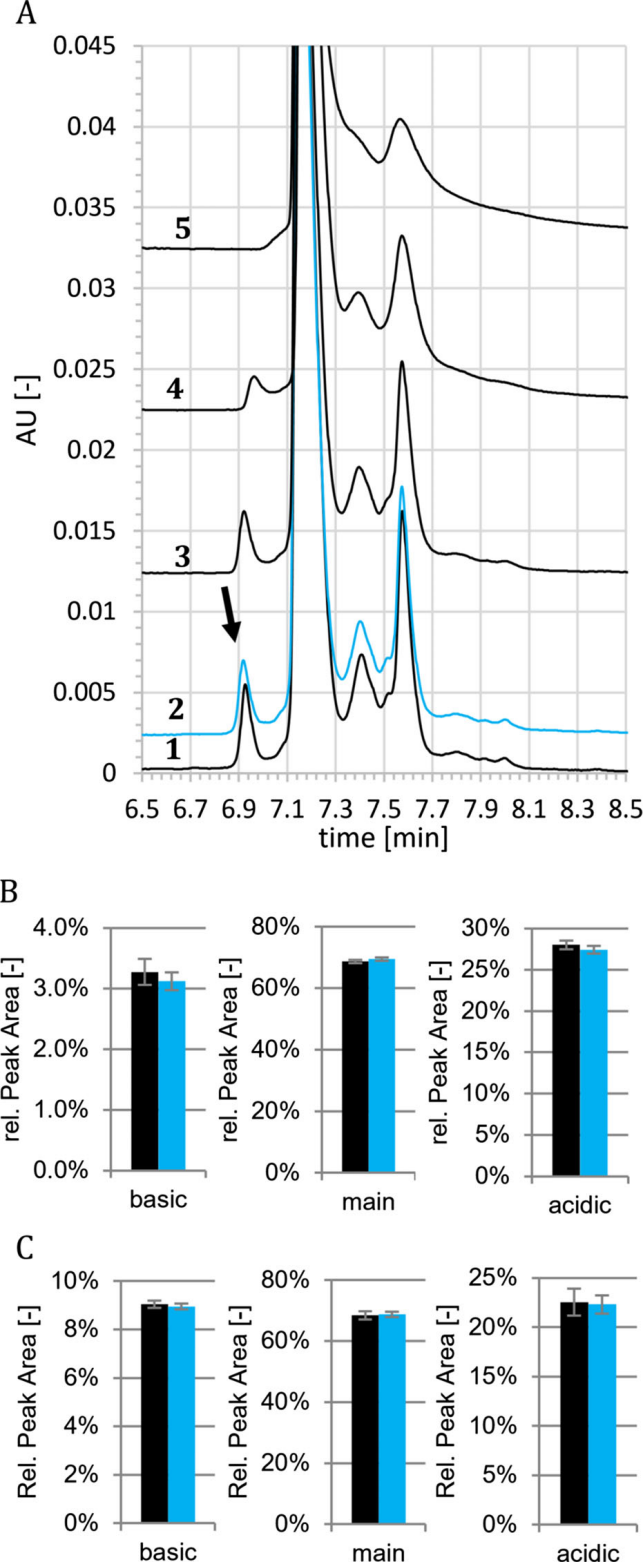
GdnCl/phosphate buffer rinsing significantly improves the reproducibility of charge heterogeneity profiles. (A) After initial mAb1 injections (line 1), mAb1Ag was injected 36 times followed by five mAb1 injections (line 2; first separation of mAb1 after 36 mAbAg1 runs). Afterwards, additional 24 mAb1Ag injections were performed and finally five mAb1 runs completed the sequence (line 3; first separation of mAb1 after 60 mAbAg1 runs). The experiment was repeated with the same capillary resulting in total 120 injections of mAb1Ag (line 4; first separation of mAb1 after 96 mAbAg1 runs; line 5; first separation of mAb1 after 120 mAbAg1 runs). Rinsing with 1.2 M GdnCl; 50 mM Na_2_HPO_4_ was performed for 10 min at 60 psi before each injection. This procedure enables reproducible charge profiles for about 40 antigen injections. For a better comparison of the peak profiles, the x‐axis has been rescaled. Injections of mAb1 (B) and mAb2 (C) (without antigen) rinsed with HCl for 1 min (black bars) were compared with injections preceded by 1.2 M GdnCl rinsing for 10 min (blue bars). Displayed error bars represent standard deviations of 12 measurements on two devices. The datasets show low standard deviations and no significant differences between the relative peak areas for both mAbs.

In order to measure the efficiency of different rinsing procedures, a simple assay was established, that consists of three steps. Before each injection the respective rinsing procedure was applied. In the first step, injections of mAb1 at 3 mg/mL were performed in a new capillary, serving as reference that describes the unaffected CZE system, i.e. no adsorbed protein at the inner capillary wall present. In the second step, at least ten injections of pure mAb1Ag (1.8 mg/mL) were made, simulating the “contamination” of the capillary with the component that tends to adsorb strongly to the capillary wall as it is assumed to appear during regular separation. During the third step, mAb1 at 3 mg/mL was injected again to test for the effect of protein adsorption and the efficiency of the applied rinsing step(s), respectively. For this test, the obtained electropherogram of mAb1 (after rinsing) is compared to the initially obtained one for mAb1 (Fig. 2). All injections of pure mAb1Ag were preceded by pre‐running rinsing steps of different design (described later in detail). While HCl as rinsing agent has shown to deliver highly reproducible results for diverse mAbs 9, in presence of antigen used in this study the cleaning is insufficient as demonstrated by still strongly influenced peak profiles after rinsing. The missing charge species in the profile (indicated by the black arrow in Fig. 2) seems to have a high adsorbance trend to the inner capillary wall. The intensity of other peaks decreased as well. These results indicate that the antigen may bind to the inner capillary wall and cannot get desorbed during HCl rinsing. When the antibody mAb1 is injected, the dynamic capillary coating with TETA may not be properly formed anymore and the absorbed antigens may serve additionally as nucleation point for even further unspecific adsorption of sample to the inner capillary wall. This hypothesis is supported by the observation, that the reduced resolution is also obtained if the adsorbed antigen is not specific to the antibody analyzed (data not shown). Accordingly, alternative rinsing approaches have been designed to reduce both, the assumed adsorption of sample material to the inner capillary wall and the resulting impact on the obtained CZE profile for analyzed mAbs. Best results, as summarized in Table 1, were achieved with solutions with high salt concentrations as rinsing agents. Following the guidance of the *Hofmeister series*, which describes the effects on solubility of proteins by salts 28, various salts in different concentrations were tested. In order to test efficiency and limits of the established rinsing procedure to reduce inner capillary wall adsorption, step 2 of the assay was extended to up to 120 injections of antigen. Best results in terms of reproducibility were obtained with different guanidine salts at 1.2 M in 50 mM Na_2_HPO_4_ (Figure 3), potentially due to the combination of a strong salting‐in agent (Guanidinium), including denaturation of the adsorbed protein and resulting better solubility, with a salting‐out agent (Phosphate). Further detailed investigation on the background of the observed effects was not in scope of this paper. In the optimized condition, the capillary was flushed with 1.2 M guanidine HCl in 50 mM Na_2_HPO_4_ (later called GdnCl/phosphate buffer rinse) instead of 0.1 M HCl for 10 min prior to each run. Results for mAb1 are now reproducible for up to 36 injections of mAb1 antigen (Figure 3). Similar results were observed for mAb2 and mAb2Ag (data not shown). In order to qualify that the alternative rinsing procedure does not impact the peak pattern in comparison to the former HCl rinsing, mAb1 or mAb2 were injected either after HCl rinse or GdnCl/phosphate buffer rinse (10 min each). Experiments were performed on two different devices and each sample was injected six times, i.e. each rinsing procedure was repeated 12 times. No significant differences were found between the two rinsing conditions, which demonstrates that the new rinsing procedure is reproducible and has no impact on the relative area percentages of the main peak, the basic peak and the acidic peaks of mAb1 (Fig. 3B) and mAb2 (Fig. 3C). Although a sustainable and enduring removal of antigens cannot be fully achieved and frequent capillary changes are required for long‐term reproducibility, this procedure provides robust results for sequences of typical length for QC applications.

Beside the GdnCl/phosphate buffer rinsing, other alternative rinsing approaches have been tested and did not show sufficient inner capillary wall cleaning as indicated by electrophoretic profile reproducibility. Harsher acidic (0.5 M and 1 M HCl) and combination of acidic and basic rinsing (0.1 M; 0.5 M 1.0 M HCl and NaOH) did not improve but decreased resolution in subsequent runs. Rinsing with SDS in buffered solution led to a complete loss in resolution for all small peaks, i.e. just one broad peak surrounding the main peak was detected (data not shown). It is assumed that SDS binds to the antigen, but is not able to dissolve it completely so that SDS remains in the capillary as well, inducing a poor separation. Organic solvents (methanol, ethanol, isopropanol, and acetonitrile) were tested; each at concentrations v/v % of 10, 20, 30, and 40%. Although all of them have a positive effect on overall peak recovery, the basic peak was still missing after 10 injections of antigen (data not shown). All these results support further the hypothesis of hydrophobic interactions of the antigen with the inner capillary wall.

Apparently, the BGE used here, is not suitable to prevent adsorption events for some kind of proteins. Antigens may bind to the inner capillary wall and accumulate within consecutive runs. These clusters may then serve as nucleation points for unspecific binding of mAbs, thus leading to adsorption. This effect may continue with each run, finally resulting in decreased peak recovery (Fig. 3). Our results show that the high ionic strength of selected salts apparently slows down the assumed adsorption events. In CE literature, usually a high salt concentration is recommended for the BGE 29, but less attention was paid to salts as rinsing agents. Kunkel et al. have tested saturated NaCl and KNO_3_ solutions as rinsing agents, but reported unsatisfactory results 30. In material sciences efforts have been made to characterize adsorption and desorption at silica surfaces, e.g. the influence of pI and ΔG determination 31, 32. Docoslis et al. have characterized GdnCl, HCl, acetic acid, PBS, GdnSCN, and Urea for their ability to desorb human serum albumin from silica surfaces 33. Best results were achieved with 4.5 M Urea at high pH and high temperature. PBS, GdnCl, and GdnSCN were less effective. Despite limited comparability between the conditions in this study and the results presented here, the observations are similar.

### 3.2 Charge heterogeneity profiling of co‐formulated mAbs

In ACE, CZE separation is combined with simultaneous protein‐ligand interactions 17. That increases specificity for important compounds even in complex samples like antibody co‐formulations. The binding of a ligand has to change charge and/or the hydrodynamic radius of the analyte in order to enable a binding related electrophoretic separation. Binding equilibria can be established in different ways, strongly depending on the binding constants. For tight binding events pre‐incubation of the protein and its ligand is suitable since the complex should be stable (inert) throughout CZE separation. Although first separations after antigen/antibody pre‐incubation looked promising, unspecific binding between mAb1 and mAb2 antigen or between mAb2 and mAb1 antigen occurred (data not shown). In order to minimize interaction time between mAb and antigen, a FTPF approach was tested. Goal of this approach was the minimization of unspecific binding and full recovery of resolution. For FTPF, the mAbs and the selected ligand are injected as two separate plugs into the capillary. The experiment necessitates that mAbs and ligand have different electrophoretic mobilities, so that one will pass the other in the capillary during electrophoresis. Interaction between mAbs and antigen then takes place, when one zone migrates through the other. Thus, the binding reaction occurs inside the capillary within a short time frame (approx. 1 min) which should minimize slow unspecific reactions. Further, the consumption of (potentially rare and complex to produce) antigen per analysis is significantly reduced.

**Figure 4:**
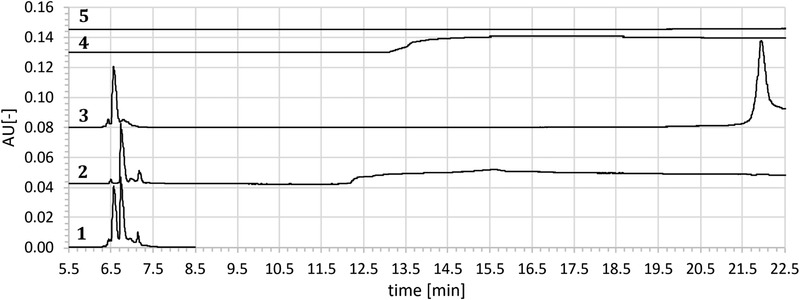
ACE‐Analysis of mAb1/mAb2 co‐formulation: Addition of mAb1Ag and mAb2Ag to the co‐formulation leads to a shift of mAb1 and mAb2 respectively. 1) co‐formulation of mAb1/mAb2 10 mg/mL. 2) co‐formulation 10 mg/mL injected with mAb1Ag leading to a pure peak pattern of mAb2 and the mAb1‐mAb1Ag complex. 3) co‐formulation 10 mg/mL injected with mAb2Ag leading to a pure peak pattern of mAb1 and the mAb2‐mAb2Ag complex. 4) injection of pure mAb1Ag 5) injection of pure mAb2Ag.

Preliminary tests demonstrated that mAb1 antigen and mAb2 antigen migrate almost approximately two times slower than mAb1 and mAb2. Therefore, the antigen was injected first (0.5 psi for 10 s), followed by the mAb plug (0.3 psi for 3 s) and BGE (0.3 psi for 3 s). Between runs, a GdnCl/phosphate buffer rinse was performed (see above). In capillary mAb/antigen interaction then led to the isolation of unbound charge species with sustained resolution (Fig. 4). The shifted charge species of the bound mAb are visible at later migration times. However, resolution of the shifted mAb‐Ag complex is compromised and hence always requires a second experiment with the other antigen for charge heterogeneity profiling. Then, the other antibody is shifted which keeps the resolution of the antibody that was shifted in the first experiment. Although the shifted antibody is not resolved, its presence still confirms the underlying principle of this FTPFACE approach, i.e. the formation of a stable mAb‐Ag complex.

**Figure 5:**
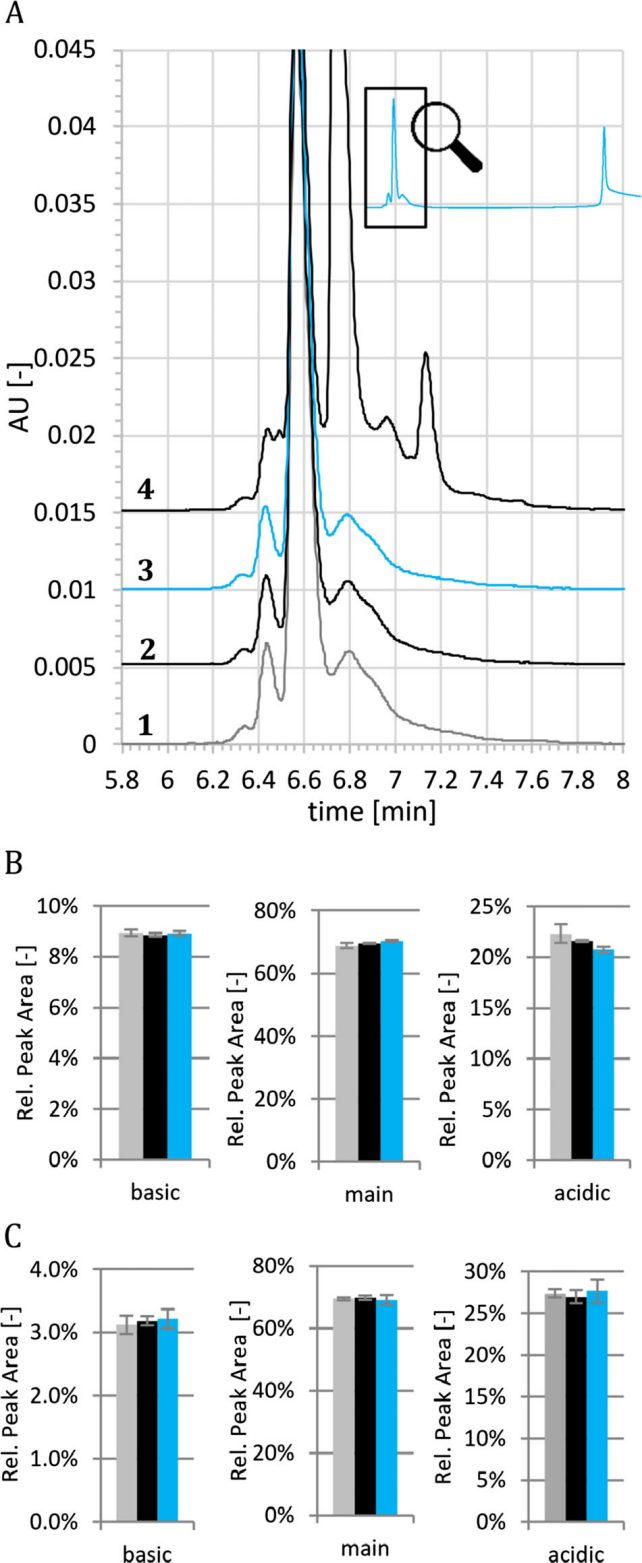
Comparison of FTPFACE with different control runs. (A) *Line 1 (grey)*: Standard mAb2 CZE separation without antigen and without pre‐running injection plug preceded by HCl rinse. *Line 2 (black)*: mAb2 CZE separation with pre‐running PBS injection plug but without antigen preceded by GdnCl rinse. *Line 3 (blue)*: mAb1/mAb2 co‐formulation with pre‐running mAb1‐antigen containing PBS injection plug (complete FTPFACE approach) preceded by GdnCl rinse. *Line 4 (black)*: CZE separation of the mAb1/mAb2 co‐formulation with pre‐running PBS injection plug but without antigen preceded by GdnCl rinse. *Insert*: Unzoomed view of line 3. The second peak contains the shifted mAb1–mAb1Ag complex. For a better comparison of the peak profiles, the x‐axis has been rescaled. (B) relative corrected peak area results for mAb2; *grey*: mAb2 sample without antigen and without second injection plug; *black*: mAb2 sample without antigen but with pre‐running PBS buffer plug; *blue*: mAb1/mAb2 co‐formulation sample with complete FTPFACE, i.e., with mAb1‐antigen in the pre‐running PBS buffer plug. The error bars show standard deviations of 12 measurements on two devices, which demonstrate excellent reproducibility and confirms full comparability of FTPFACE and standard CZE. The bar colors correspond to the separations shown in A. (C) same as (B) for mAb1. All separations were performed with pre‐running GdnCl/phosphate buffer rinsing.

In order to verify the reproducibility and comparability of the FTPFACE approach with a standard CZE analysis, FTPFACE results for the co‐formulation were compared with the results from different control runs: 1) a standard CZE analysis of either mAb1 or mAb2 and their co‐formulation and 2) FTPFACE injections of either mAb1 or mAb2 using PBS instead of antigen. A visual comparison of the control runs with FTPFACE of the co‐formulation shows a complete recovery of either mAb1 or mAb2 charge species and no interference anymore between mAb1 and mAb2 (exemplarily shown for mAb2 in Fig. 5A, not shown for mAb1). In order to evaluate the potential impact of a second (antigen) plug and third BGE plug on the area percent results all experiments were performed six times on two devices (*n* = 12 injections in total). The obtained variation was low (relative standard deviation far below 5%), showing excellent reproducibility of the method for mAb2 (Fig. 5B) and mAb1 (Fig. 5C). Additionally, no significant area percent changes occurred between standard CZE and the different FTPFACE injection modes. This demonstrates a complete comparability of standard CZE 8 with FTPFACE. Experiments with aged material of mAb1 an mAb2 (one month at 40°C) were conducted as well in order to proof usability for QC testing (Supporting Information Fig. S1 and S2). A complete shift of all species of stressed mAb1 and mAb2 sample was obtained. However, these results might not be transferrable to mAbs that have a strong decay in binding efficacy in case they are stressed.

FTPFACE was introduced a long time ago for the estimation of binding constants 23. However, only very few groups have combined this technique with mAbs. For example Grubor et al. 34 used the high specificity of mAbs for binding one of the enantiomers of an inseparable racemate. PFACE was also used for the monitoring of oligosaccharide epitopes in therapeutical mAbs. 35

In summary, FTPFACE is easy to perform and runs on standard CE equipment. Furthermore, it is fast, robust and highly specific for single charge species of complex antibody mixtures. It is fully suitable for GMP applications and should always be considered for co‐formulated antibodies that are too similar for conventional charge heterogeneity profiling.

## 4 Concluding remarks

Charge heterogeneity profiling is important for the QC testing of biopharmaceuticals. As novel drug designs are highly complex, QC methods have to be continuously improved. In this work, ACE was tested for this purpose and applied for antibody specific charge heterogeneity profiling of co‐formulated mAbs.

The main challenge of ACE method development was a lack of run‐to‐run reproducibility that caused a poor peak recovery and resolution of species. This was assumed to be related to antigen adsorption to the inner capillary wall. Since it was favorable to maintain well established and optimized separation conditions, the main focus of this study was on enhancing protein desorption from the inner capillary wall before each analysis. Chaotropic guanidine hydrochloride, an agent that cuts H–bonds and denatures protein structures, in combination with kosmotropic phosphate buffer was identified to significantly increase reproducibility. It could be attributable to the weakening of adverse interactions by charges and hydrophobic parts of the molecule in parallel that then may allow a better release from the inner capillary surface. Extensive rinsing with guanidine in combination with phosphate preserves comparable peak patterns with excellent peak recoveries for more than 30 antigen injections which is sufficient for QC routine analytics. After an analysis bare fused silica capillaries can be easily exchanged.

It has been successfully demonstrated that FTPFACE analysis of the mAb1/mAb2 co‐formulation + mAb1Ag leads to the same peak pattern as a standard CZE separation of mAb2, whereby GdnCl/phosphate buffer rinsing safeguarded an excellent reproducibility for up to 36 runs. The same was found for mAb1. A study with aged samples has shown that the method is applicable for stability testing. Due to several reasons, the co‐formulation of mAb1 and mAb2 can be regarded as a difficult sample for this kind of analysis. First of all, mAb1 and mAb2 are very similar and have strongly overlapping profiles. In addition, mAb1 and mAb2 antigens strongly impede reproducibility, presumably by interaction with the inner capillary. This requires an elaborated capillary rinsing procedure. In spite of this, FTPFACE was very successful which clearly demonstrates the generic potential of this approach and its universal applicability for many samples of this type that may even be less ambitious than the sample investigated in this study. It was possible to outline and optimize critical parameters for FTPFACE that will support future method development for other samples.

In conclusion, this work has presented a fully GMP QC applicable FTPFACE approach, which enables charge heterogeneity profiling of complex mAb mixtures. While antibody co‐formulations may play an increasingly important role in the future of many kinds of therapies, this technique allows an economical and appropriate charge heterogeneity profiling of these complex biopharmaceuticals.

*The authors have declared no conflict of interest*.

## Supporting information

Supporting material
