# Supplementary material for: Application of affinity capillary electrophoresis for charge heterogeneity profiling of biopharmaceuticals
Source: Electrophoresis. 2019 Oct 8;40(22):3014–22. doi: 10.1002/elps.201900233 (PMC6900010; doi:10.1002/elps.201900233)
Supplement: Supplementary file 1 — Supporting material [file ELPS-40-3014-s001.docx]

**Supportive Information**

**Application of Affinity Capillary Electrophoresis for Charge Heterogeneity Profiling of Biopharmaceuticals**

Andrei Hutanu^(1)*^, Steffen Kiessig^(1)^, Andrea Bathke^(1)^, Rolf Ketterer^(1)^, Sonja Riner^(1)^, Jan Olaf Stracke ^1)^, Markus Wild^(1)^, Bernd Moritz^(1)^

(1) F. Hoffmann-La Roche Ltd, Grenzacherstr 124, 4070 Basel, Switzerland

*Corresponding author: e-mail: andrei.hutanu@roche.com

*Keywords:* Affinity Capillary Electrophoresis, monoclonal antibody co-formulation, charge heterogeneity testing, flow-through partial filling

Figure S1


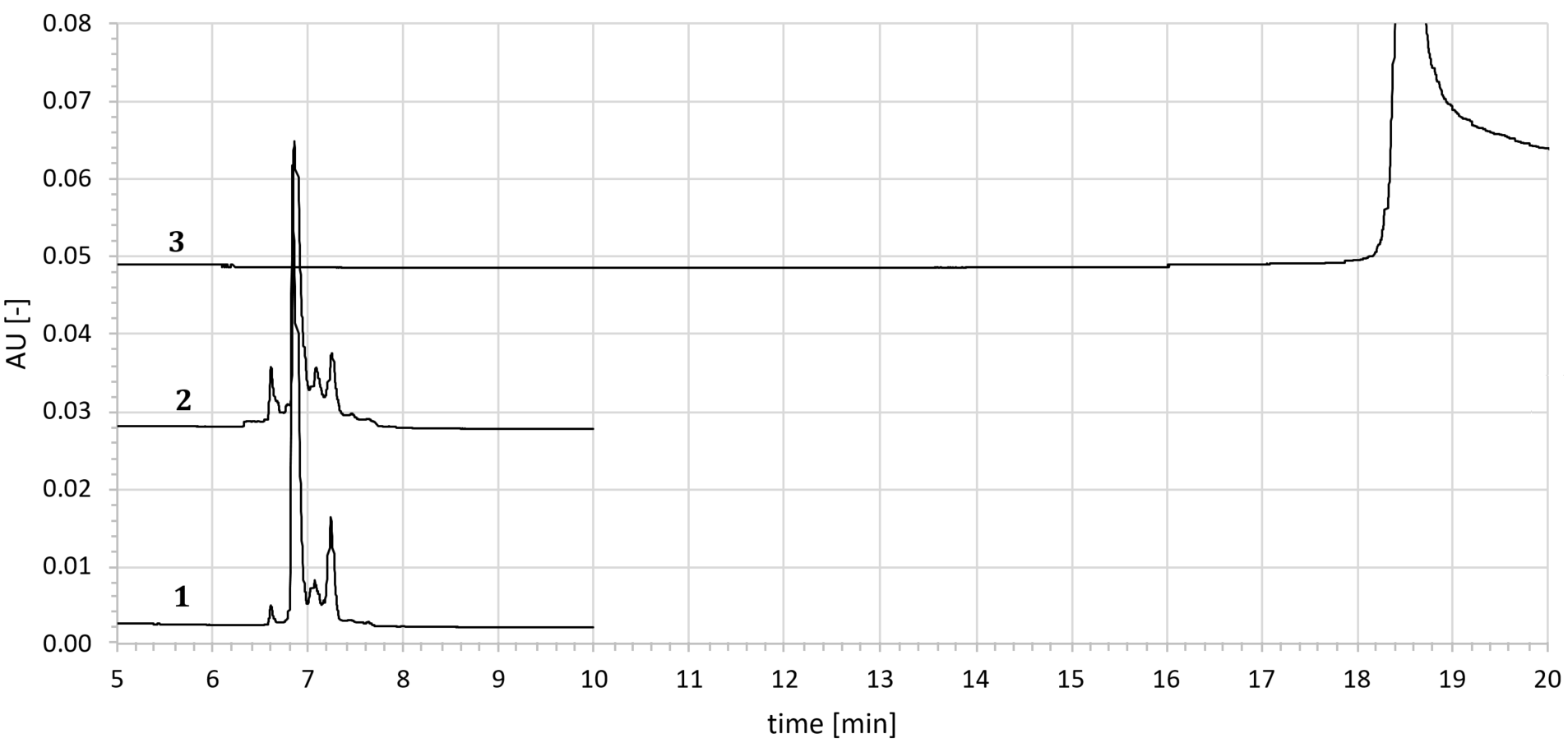


Figure S1: FTPFACE with stressed mAb1 samples. *Line 1:* mAb1 CZE separation with pre-running PBS injection plug but without antigen (5 mg/mL). *Line 2:* stressed mAb1 (1 month at 40°C) CZE separation with pre-running PBS injection plug but without antigen. *Line 3:* stressed mAb1 (1 month at 40°C) FTPFACE separation with pre-running mAb1Ag injection plug resulting in a mAb1-mAb1Ag complex.

Figure S2


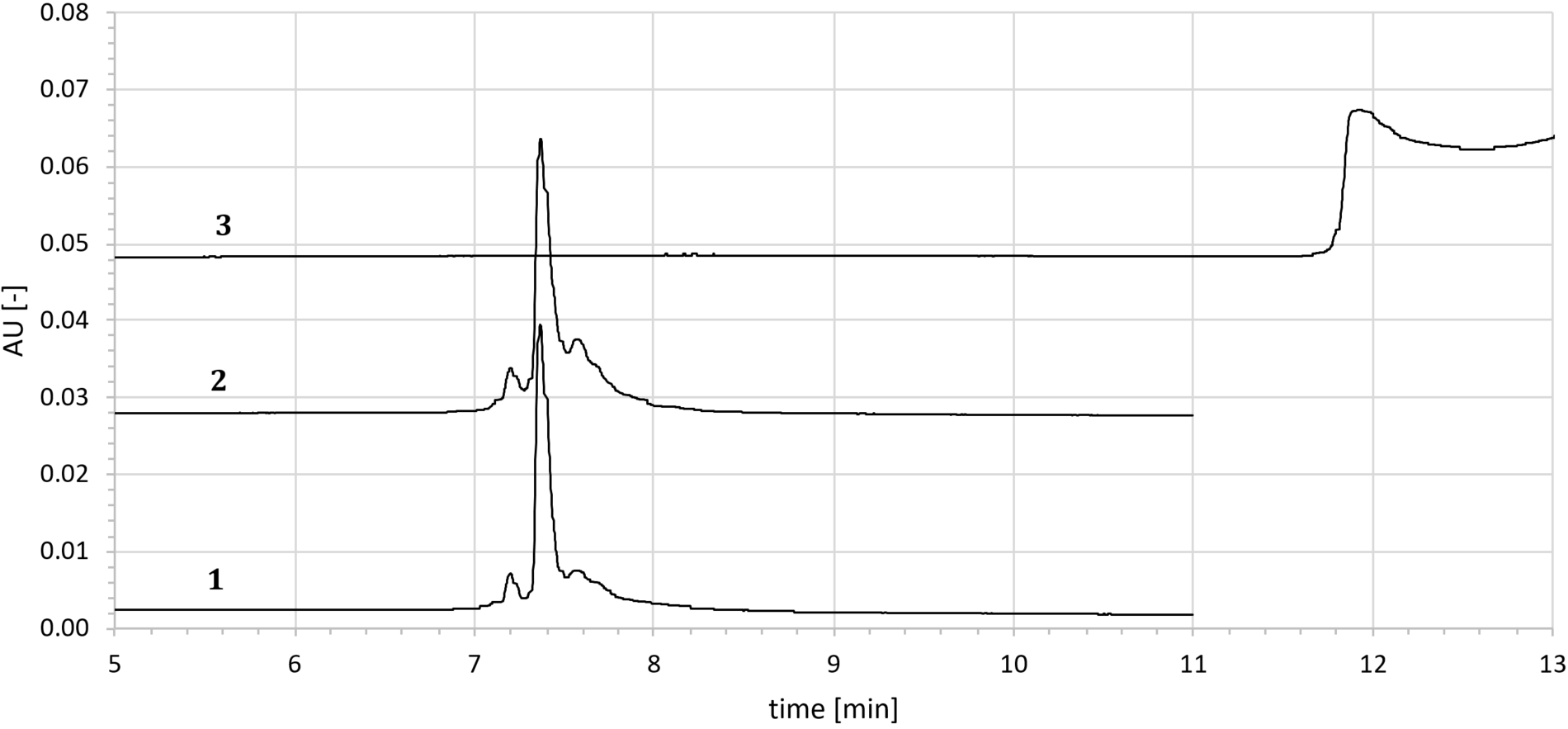


Figure S2: FTPFACE with stressed mAb2 samples. *Line 1:* mAb2 CZE separation with pre-running PBS injection plug but without antigen (5 mg/mL). *Line 2:* stressed mAb2 (1 month at 40°C) CZE separation with pre-running PBS injection plug but without antigen. *Line 3:* stressed mAb2 (1 month at 40°C) FTPFACE separation with pre-running mAb2Ag injection plug resulting in a mAb2-mAb2Ag complex.
